# Association Between a Tri-allelic Polymorphism in the Estrogen Metabolism Oxidoreductase NRH:Quinone Oxidoreductase 2 Gene and Risk of Breast Cancer by Molecular Subtype

**DOI:** 10.3389/fgene.2021.658285

**Published:** 2021-03-12

**Authors:** Jiao-Qun Zhou, Si-Yuan Zhu, Ye He, Ke-Da Yu

**Affiliations:** ^1^Department of General Surgery, The First People’s Hospital of Fuyang, Fuyang, China; ^2^Department of Breast Surgery, Fudan University Shanghai Cancer Center and Cancer Institute, Shanghai Medical College, Fudan University, Shanghai, China; ^3^Department of Radiotherapy, Fudan University Shanghai Cancer Center, Shanghai Medical College, Fudan University, Shanghai, China

**Keywords:** NQO2, SNP, breast cancer, luminal-like, molecular subtype

## Abstract

**Background**: We hypothesized that NRH:quinone oxidoreductase 2 (NQO2) is a candidate susceptibility gene for breast cancer because of its known enzymatic activity on estrogen-derived quinones. A tri-allelic polymorphism in the NQO2 gene might be associated with the risk of luminal-like breast cancer.

**Methods**: In this case-control study, 2,865 women were recruited, including 1,164 patients with pathologically confirmed breast cancer and 1,701 cancer-free controls. The tri-allelic genetic polymorphism (I-29, I-16, and D alleles) was genotyped by a polymerase chain reaction and restriction fragment length polymorphism (RFLP)-based assay. Because the I-16 allele frequency is rare (approximately 1.0%), individuals carrying the I-16 allele were excluded from the analysis. Breast cancer subtypes were classified according to ER, PR, HER2, and grade.

**Results**: In the association analysis of allele, an increased risk of breast cancer is associated with I-29 allele [82.5% in case group and 79.0% in the control group; odds ratio (OR), 1.25; 95% CI, 1.09–1.43, compared with D allele, *p* = 0.0015]. In the association analysis of genotype, the I-29-containing genotype was significantly correlated with breast cancer under a dominant model (adjusted OR, 1.31, 95% CI, 1.12–1.54, *p* = 0.001). Moreover, in the subtype analysis, there was a significant association of the I-29/D polymorphism with luminal-like breast cancer (adjusted OR, 1.54, 95% CI, 1.22–1.94, *p* = 0.001 for luminal-A disease; adjusted OR, 1.37, 95% CI, 1.06–1.76, *p* = 0.014 for luminal-B disease) but not with HER2-enriched or triple-negative subtypes.

**Conclusion**: The tri-allelic polymorphism in the NQO2 gene is associated with breast cancer risk, especially for the luminal-like subtype. Our findings provide a new piece of molecular epidemical evidence supporting the hypothesis that estrogen and its metabolites are carcinogens of luminal-like breast cancer. Further external validation studies are needed.

## Introduction

Estrogen and its metabolites may play an essential role in breast carcinogenesis (Yager and Davidson, 2006). Physiological metabolism of estrogen and estrogen-related environmental factors may lead to chronic exposure to estrogen-derived quinone/semiquinone, which was recognized as putative carcinogens. Quinone oxidoreductase is a phase II detoxification enzyme that can neutralize carcinogens and catalyze the reduction of quinone metabolism. Two isoforms of quinone oxidoreductase have been identified, namely NAD(P)H: quinone oxidoreductase 1 (NQO1) and NRH:quinone oxidoreductase 2 (NQO2; Jaiswal, 2000).

Gaikwad et al. (2009) discovered for the first time that NQO2 could catalyze the reduction of estrogen quinone and act as a detoxification enzyme. In addition, they also proved that NQO2 is faster than NQO1 in reducing estrogen quinone. It is logical to assume that NQO2 is a candidate gene for breast cancer susceptibility. Previously, we conducted a case-control study and found that NQO2 is a susceptibility gene for breast cancer, and it confers specific protection against wild-type p53 breast cancer (Yu et al., 2009c). It is worth noting that the TP53 gene is often a mutant in triple-negative breast cancer (TNBC), but less mutated in luminal-like disease (Jiang et al., 2019). The precise mechanism of how NQO2 affects tumorigenesis of breast cancer is yet unknown and to study. However, some studies are revealing the potential mechanism of NQO2 in other cancers. For instance, NQO2 has a role in controlling AKT/GSK-3β/cyclin D1 and highlights the involvement of NQO2 in the degradation of cyclin D1 (Hsieh et al., 2012).

Among the genetic variations of the NQO2 gene, the tri-allelic genetic polymorphism of I-29/I-16/D is the most studied genetic variation. It is located in the NQO2 promoter region, plays an important role in gene expression, and is related to Parkinson’s disease (Harada et al., 2001; Wang et al., 2008). It is worth noting that Wang et al. (2008) have proved that the previously reported I-29/D polymorphism is indeed tri-allelic, consisting of 29-bp insertion (I-29), 29-bp deletion (D), and 16-bp insertion (I-16). We have reported that the frequencies of the I-29 allele and D allele are different between breast cancer cases and healthy controls, and that I-29 is a risk allele for breast cancer. In the era of breast cancer subtypes, we would like to study further the relationship between this tri-allelic genetic polymorphism in the NQO2 gene and the risk of breast cancer by molecular subtypes.

## Materials and Methods

### Study Subjects

In this case-control study, 2,865 unrelated women were recruited, including 1,164 patients with pathologically confirmed primary breast cancer and 1,701 women as cancer-free controls. Breast cancer patients came from the Fudan University Shanghai Cancer Center and the Fuyang First People’s Hospital. Patients with a previous history of other cancers or metastatic breast cancer were excluded. All selected cases have been tested for germline mutations in BRCA1, BRCA2, BRIP1, and PALB2 (Li et al., 2008; Lang et al., 2017), and no deleterious genetic mutation was found.

In terms of controls, 1,701 healthy women were selected from a community-based breast cancer screening project initiated by Fudan University Shanghai Cancer Center and Shanghai Municipal Center for Disease Control and Prevention. This cohort program screened more than 20,000 women (aged between 35 and 75 years) for breast cancer. Breast cancer screening methods include physical examination, ultrasound, and mammography (Yu et al., 2009c; Mo et al., 2013). Women with a previous history of cancer were also excluded from the control group.

This study was approved by the Ethics Committee of Shanghai Cancer Center of Fudan University and Fuyang First People’s Hospital, and each participant signed an informed consent form.

### DNA/RNA Preparation

PCR was performed as described previously (Yu et al., 2009b). According to the manufacturer’s protocol, Gentra’s PureGene DNA Purification Kit (Gentra Systems, United States) was used to extract genomic DNA from 3 to 5 ml of peripheral blood leukocytes of the subject and then stored at −20°C.

### Genotyping

The tri-allelic genetic polymorphism was genotyped by a PCR and restriction fragment length polymorphism (RFLP) based assay (Yu et al., 2009a,c). In brief, we performed PCR (primers: sense 5'-CTGCCTGGAAGTCAGCAGGGTC-3'; antisense 5'-CTCTTTACGCAGCGCGCCTAC-3') and used a lengthy electrophoresis-based method to distinguish I-29, I-16, and D allele. Since there is only a 13 bp difference between the I-29 allele and I-16 allele, it is difficult to separate by general electrophoresis. Therefore, we used an RFLP-based method and used restriction enzyme Ava I to discriminate between the I-16 and I-29 alleles. The I-29 allele was digested by Ava I into 250 and 40 fragments, while I-16 was not digested. Use a sufficient amount of restriction enzyme to cleave the PCR amplicon completely. Two research assistants (J-QZ and S-YZ) independently checked the gel pictures and repeated the assays if they did not reach a consensus on the genotype. In addition, 10% of samples were randomly selected for repeated RFLP assay of the two polymorphisms, and the results were 100% concordant.

### Breast Cancer Molecular Subtype

Pathologists in the Department of Pathology determine the status of estrogen receptor (ER), progesterone receptor (PR), and HER2. A positive ER or PR requires 1% or more of tumor cells to be immunoreactive (Hammond et al., 2010). Patients with an equivocal expression of HER2 protein (immunohistochemistry 2+) will undergo the FISH test for HER2 gene amplification. Due to the lack of Ki67 information in some cases, molecular subtype was categorized as following (Arvold et al., 2011): luminal-A, ER/PR-positive, HER2-negative and grade I-II; luminal-B, ER/PR-positive, HER2-positive or grade III; HER2-enriched, ER and PR-negative, HER2-positive, any grade; and TNBC, ER, PR, and HER2-negative, any grade.

### Statistical Analysis

Association analysis is performed using Pearson’s *χ*^2^ test or Fisher’s exact test when appropriate. An odds ratio (OR) with 95% CIs were also determined. We used Student’s *t*-test or Mann-Whitney test to compare continuous variables between the two groups, and used logistic regression to analyze the association between genetic variation and breast cancer risk. A value of *p* ≤ 0.05 is considered statistically significant. Statistical analysis was performed using Stata version 16 (StataCorp LLC) and SPSS version 19 software (SPSS, Chicago, IL).

## Results

### Basic Information of Cases and Controls

Cases and controls were comparable in age (mean age ± SD: 48 ± 10 years for case vs. 47 ± 9 years for control, *p* = 0.6), age at menarche (14 ± 2 years for case vs. 14 ± 2 years for control, *p* = 0.8), and body mass index (23.3 ± 3.1 for case vs. 22.6 ± 3.3 for control, *p* = 0.2). However, compared with the healthy control group, breast cancer patients had more family history of breast cancer (13.5% for case vs. 7.8% for control, *p* < 0.001; Table 1).

**Table 1:** Basic information of the breast cancer cases and the healthy controls.

Characteristics		Control (*N* = 1701)	%	Case (*N* = 1,164)	%	*p*
Age (years)	Mean, SD	47	9	48	10	0.6
Age at menarche (years)	Mean, SD	14	2	14	2	0.8
Body mass index	Mean, SD	22.6	3.3	23.3	3.1	0.2
Family history of breast cancer	No	1,568	92.2	1,007	86.5	<0.001
	Yes	133	7.8	157	13.5	
Menopausal status	Pre/perimenopausal	1,014	59.6	738	63.4	0.041
	Postmenopausal	687	40.4	426	36.6	
Breast cancer subtype	Luminal-A	-	-	435	37.4	
	Luminal-B	-	-	341	29.3	
	HER2-enriched	-	-	116	10.0	
	Triple-negative	-	-	272	23.4	
Genotype of NQO2 tri-allelic polymorphism	I-29/I-29	1,024	60.2	778	66.8	0.015
	I-29/D	568	33.4	329	28.3	
	D/D	63	3.7	36	3.1	
	I-29/I-16	31	1.8	13	1.1	
	D/I-16	10	0.6	6	0.5	
	I-16/I-16	5	0.3	2	0.2	
Allele of NQO2 tri-allelic polymorphism	I-29	2,647	77.8	1898	81.5	0.002
	D	704	20.7	407	17.5	
	I-16	51	1.5	23	1.0	

SD, standard deviation.

### Tri-allelic Genetic Polymorphism and Breast Cancer Risk

**Figure 1:**
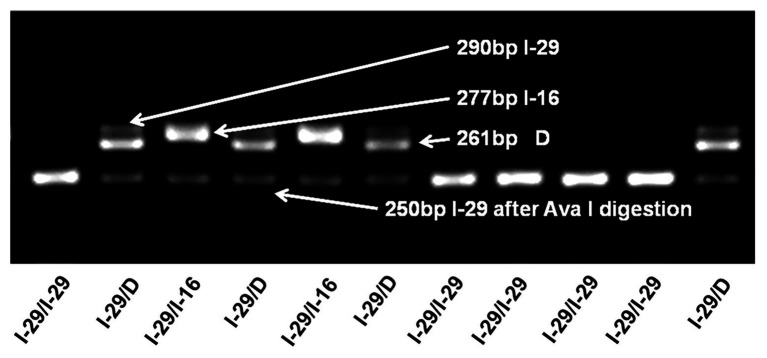
Genotyping of the tri-allelic polymorphism in NQO2. A typical gel electrophoresis plot of the I-29/I-16/D polymorphism is shown. Samples are assayed by PCR and electrophoresis. I-29 (290 bp) can be digested by Ava I into 250 and 40 bp fragments, but I-16 (277 bp) and D allele (261 bp) cannot. Correspondingly, I-29/I-29 can be completely digested, and no band is observed at 290 bp. In contrast, I-29/I-16 has two bands, one is 250 bp, and the other is 277 bp. For I-29 heterozygotes, even with enough restriction enzymes, I-29 alleles may not be completely digested, leaving a shadow band at 290 bp. This may be because Ava I cannot effectively cleave the dimeric I-29/I-16 or I-29/D complex.

Genotyping of the tri-allelic genetic polymorphism (I-29/I-16/D) was performed by a PCR and RFLP based assay (representative gel electrophoresis diagram in Figure 1). The I-16 allele frequency is very low (1.5% in the control group and 1.0% in the case group), so it is difficult to reach a sufficient statistical power to detect the association between the I-16 allele and breast cancer risk. In addition, preliminary data indicate that the biological role of the I-16 allele might be different from that of I-29. Therefore, we excluded individuals carrying the I-16 allele from further association analysis.

Then, we evaluated the frequency distribution of the I-29 allele and D allele in 1,143 cases and 1,655 controls. As shown in Table 2, the increased risk of breast cancer is associated with I-29 allele (82.5% in the case group and 79.0% in the control group; OR, 1.25; 95% CI, 1.09–1.43, compared with D allele, *p* = 0.0015). To confirm the association between I-29/D genotypes and breast cancer, we applied logistic regression analysis. The results were adjusted for age, age at menarche, menopausal status, and body mass index. The I-29-containing genotype was significantly correlated with breast cancer under a dominant model (unadjusted *p* = 0.003; adjusted OR, 1.31, 95% CI, 1.12–1.54, *p* = 0.001) rather than a recessive model (adjusted OR, 1.22, 95% CI, 0.80–1.85, *p* = 0.36).

**Table 2:** Association between NRH:quinone oxidoreductase 2 (NQO2) tri-allelic polymorphism and breast cancer risk in the whole study population.

		Control (*N* = 1,655, %)^*^		Case (*N* = 1,143, %)^*^		*p* ^ζ^	Dominant model OR (95% CI)^¶^^,^^Δ^	*p*	Recessive model OR (95% CI)^¶^^,^^Δ^	*p*
Genotype	I-29/I-29	1,024	61.9	778	68.1	0.003	1.31 (1.12–1.54)	0.001	1.22 (0.80–1.85)	0.36
	I-29/D	568	34.3	329	28.8					
	D/D	63	3.8	36	3.1					
Allele	I-29	2,616	79.0	1885	82.5	0.0015				
	D	694	21.0	401	17.5					

* individuals with the I-16 allele were excluded from the analysis.

ζ unadjusted *p*-value of two-sided *χ*^2^ test.

¶ odds ratio (OR) and 95% CI calculated by logistic regression, adjusted for age, age at menarche, menopausal status, and body mass index.

Δ For the model, homozygotes for the major allele (1/1), heterozygotes (1/2), and homozygotes for the rare allele (2/2) are coded as a continuous numeric variable for genotype (i.e., 0, 1, and 2). The dominant model is defined as contrasting genotypic groups 1/1 vs. (1/2 + 2/2), and the recessive model is defined as contrasting genotypic groups 2/2 vs. (1/1 + 1/2).

### Tri-allelic Genetic Polymorphism and Risk of Breast Cancer Subtype

Because NQO2 is an oxidoreductase of estrogen metabolism, we hypothesized that NQO2 might have a more significant protective effect in the luminal-like subtype. There were 423 cases of luminal-A, 338 cases of luminal-B, 113 cases of HER2-enriched, and 269 cases of TNBC. We observed a significant association of the I-29/D polymorphism with luminal-like breast cancer (adjusted OR, 1.54, 95% CI, 1.22–1.94, *p* = 0.001 for luminal-A disease; adjusted OR, 1.37, 95% CI, 1.06–1.76, *p* = 0.014 for luminal-B disease) but not with HER2-enriched (*p* = 0.44) or TNBC (*p* = 0.76; Table 3).

**Table 3:** Association between genotypes of NQO2 tri-allelic polymorphism and risk of breast cancer subtypes.

Genotype	Control (*N* = 1,655, %)^*^		Luminal-A case (*N* = 423, %)^*^		Luminal-B case (*N* = 338, %)^*^		HER2-enriched case (*N* = 113, %)^*^		Triple-negative case (*N* = 269, %)^*^	
I-29/I-29	1,024	61.9	302	71.4	233	68.9	74	65.5	169	62.8
I-29/D	568	34.3	108	25.5	93	27.5	34	30.1	94	34.9
D/D	63	3.8	13	3.1	12	3.6	5	4.4	6	2.2
P			<0.001^ζ^	0.001^¶^	0.045^ζ^	0.014^¶^	0.64^ζ^	0.44^¶^	0.43^ζ^	0.76^¶^
Dominant model OR (95% CI)^¶^			1.54 (1.22–1.94)		1.37 (1.06–1.76)		1.16 (0.78–1.75)		1.04 (0.80–1.36)	

* individuals with the I-16 allele were excluded from this analysis.

ζ unadjusted *p*-value of two-sided *χ*^2^ test.

¶ OR and 95%CI calculated by logistic regression, adjusted for age, age at menarche, menopause status, and body mass index in the dominant model (I-29/I-29 vs. I-29/D+D/D).

## Discussion

In the present case-control study, we observed a significant association between breast cancer risk and a tri-allelic polymorphism in the NQO2 gene. More interestingly, this genetic variation is significantly related to the luminal-like subtype but not to the HER2-enriched and TNBC subtypes. It has been proved that the risk allele, I-29, may introduce the transcription repressor Sp3 binding site and reduce the expression of NQ2, resulting in impaired estrogen metabolism (Wang and Jaiswal, 2004). Our molecular epidemiological results are consistent with previous basic research findings.

Breast cancer appears to be the consequence of both genetic and environmental influences. Several susceptible loci and genes with different penetrance rates have been identified through linkage analysis and association study. Regarding the association between NQO2 and luminal-like breast cancer, there was limited existing evidence. NQO2, as an oxidative stress-related oxidoreductase, might protect cells from oxidative damage by catalyzing the reduction of carcinogenic quinone compounds into their hydroquinone forms. Initiation of the breast, prostate, and other cancers has been hypothesized to result from the reaction of specific estrogen metabolites, catechol estrogen-3,4-quinones, with DNA to form depurinating adducts at the N-7 of guanine and N-3 of adenine by 1,4-Michael addition (Cavalieri et al., 2002). Since NQO2 reduces the production of estrogen orthoquinones, and exposure to estrogens and estrogen orthoquinones is a risk factor of breast cancer (Gaikwad et al., 2009), we hypothesized that there might be a connection between NQO2 and estrogen metabolism-relevant breast cancer, probably, the luminal-like subtype.

We hypothesized that NQO2 is a candidate susceptibility gene for luminal-like breast cancer because it has a known activity on quinone metabolites derived from estrogen. Our analysis is designed as a genetic association study using a functional polymorphism strategy. It focuses on the I-29/I-16/D variation and the results link the NQO2 gene to the susceptibility of luminal-like breast cancer. As we know, breast cancer susceptibility variations usually show heterogeneity among tumor subtypes. A recent genome-wide association study identified 32 new susceptibility loci, five of which showed associations in opposite directions between luminal and non-luminal subtypes (Zhang et al., 2020). Our findings provide a better understanding of the genetic susceptibility of luminal-like subtype and will inform the development of subtype-specific polygenic risk assessment.

There are several limitations to this study. First, our results should be validated in external populations. Second, the I-16 allele was not thoroughly evaluated in this study because the allele frequency is too low to draw convincing conclusions. Although, we did not perform an in-depth functional experiment of the I-16 allele, the available data showed that the activity of the I-16-containing promoter was significantly increased compared with the I-29-containing promoter (Wang et al., 2008). Given the unclear role of I-16 and its low prevalence, we excluded individuals carrying the I-16 allele for association analysis. Moreover, the interaction between the genetic variation and the environmental factors should be further studied. Last but not least, we did not observe any association of the I-29/D polymorphism with HER2-enriched or TNBC, and that may be because of the heterogeneous sample size. Further validation in a large sample of patients with HER2-enriched and TNBC are needed to validate our findings.

Taken together, our data indicate that the I-29 allele in the tri-allelic polymorphism of the estrogen metabolism oxidoreductase NQO2 gene is associated with the risk of breast cancer, especially for the luminal-like subtype. Our findings provide a new piece of evidence to support the hypothesis that estrogen and its metabolites are carcinogens of luminal-like breast cancer.

## Data Availability Statement

The original contributions presented in the study are included in the article/supplementary material; further inquiries can be directed to the corresponding authors.

## Ethics Statement

The studies involving human participants were reviewed and approved by the Ethics Committee of Shanghai Cancer Center of Fudan University and Fuyang First People’s Hospital. The patients/participants provided their written informed consent to participate in this study.

## Author Contributions

K-DY and J-QZ: conception and design, administrative support, and collection and assembly of data. K-DY, J-QZ, and S-YZ: provision of study materials or patients. All authors contributed to data analysis and interpretation, and manuscript writing. All authors contributed to the article and approved the submitted version.

### Conflict of Interest

The authors declare that the research was conducted in the absence of any commercial or financial relationships that could be construed as a potential conflict of interest.
